# Diaphtherin in Dental Practice

**Published:** 1893-08

**Authors:** John Berger

**Affiliations:** Görlitz, Germany


### DIAPHTHERIN IN DENTAL PRACTICE.[1]

BY JOHN BERGER, D.D.S., GÖRLITZ, GERMANY.

SOME months ago a new antiseptic was discovered by Lembach and Schleicher, of Bieberich on the Rhine, and with regard to its chemical compounds is called oxyquinaseptol or diaphtherin. It represents a light-yellow powder of slight carbolic odor, soluble in water to a certain extent. Its formula is $HO — C_9H_6NH — O — SO_2 — C_6H_4 — O — NHC_9H_6 — OH$. It is a combination of oxyquinolin and aseptol. In higher temperatures it is reduced and oxyquinolin and carbolic acid become free.

Professor Emmerich was the first to publish experiments in regard to its antiseptic properties, according to which diaphtherin has a higher character than carbolic acid and lysol. A 3 to 1000 solution of diaphtherin was able to kill the staphylococcus pyogenes aureus in a quarter of an hour, while a 5 to 1000 solution of carbolic acid or lysol had no such effect in an equal period. A 1 to 1000 solution of diaphtherin was sufficient to destroy the Koch's bacilli of Asiatic cholera in about ten minutes; an equally strong solution of lysol could not destroy them in a period of three-quarters of an hour. The bacilli of diphtheria and some other pathogenic bacteria were destroyed by a solution of 2 to 1000 in ten minutes.

Clinical experiments with this remedy were made by Dr. Kronacher, who used diaphtherin in his surgical clinic in extirpation of carcinoma mammæ, epithelioma, and other tumors, various abscesses, drainages, fistulæ, etc. It always gave excellent results, and did not affect the hands of the operator similarly to carbolic acid. He never discovered any infection of the wounds, and claimed it as an excellent antiseptic without caustic properties. Nevertheless, it penetrates the tissue and disinfects not only the surface of

---

[1] Read at the annual meeting of the Society of the Alumni, Department of Dentistry, University of Pennsylvania, May 9, 1893, by Dr. W. E. Christensen, Philadelphia.

the wound, but also its deeper layers. Any injurious effect on the organism is absolutely excluded.

In this way diaphtherin is one of the best antiseptics ever introduced, and it did not take long before it was used in dental practice. It has been a need in dentistry to have an antiseptic that would be strong enough to destroy micro-organisms in decayed teeth without discoloring properties or other injurious effects upon the tissue. Carbolic acid is not strong enough to penetrate perfectly-curved root-canals which could not be drilled and filled perfectly to the foramen apicale. Corrosive sublimate is valuable for this, but it discolors the tooth-substance, and therefore never should be used, at least not in the front teeth. Iodoform, according to Dr. Miller, of Berlin, and others, is limited as an antiseptic, and has a very disagreeable odor, to many persons almost insupportable. Diaphtherin is a very strong antiseptic, with but a very faint odor, not unlike that of carbolic acid, and has no corrosive properties and does not discolor the tooth-substance. Dr. Hamecher, of Plauen, was the first who tried it in dental practice, and he found it to be of exceedingly great value in the treatment of root-canals. In one experiment he imperfectly removed the *débris* of a putrescent pulp, and then filled the whole pulp-chamber with a thick paste of diaphtherin and water, and inserted the filling without any disagreeable reaction following.

Even in cases where pericementitis had taken place without forming a fistula, he had most satisfactory results in filling them at the first visit of the patient. He always used diaphtherin in a solution of 1 to 100 in the treatment of fistulæ, empyemas, antri Highmori, various extractions, etc. When applied it generally causes a burning sensation, but the patient has no subsequent suffering from any changes of the mucous membrane of the mouth.

Some time ago I began using diaphtherin in my own dental practice, and I have found it to be an antiseptic to which no other preparation is equal. I will describe a few cases in which I used it.

I. Patient, lady of about twenty-six; second molar tooth. After application of arsenous acid twenty-four hours, on removing the portio coronalis of the pulp it was followed by a very disagreeable bleeding. The canals were narrow and curved. I filled the pulp-chamber with a paste made of diaphtherin and oil of cloves, and closed the cavity with cement. The tooth gave no further after-trouble.

II. Patient, lady of about thirty; bicuspid tooth with inflamed pulp and slightly inflamed pericementum. After having removed the pulp and cleaned the canals, I filled the roots at once with a mixture of diaphtherin and carbolic acid, and closed the cavity with cement. The tooth became comfortable immediately, and has so remained. Several other cases similar to these, in incisors, cuspids, and bicuspids, have all given most excellent results. In one only can I report a failure: patient, young man of about nineteen; upper first molar with chronic pericementitis. At the request of the patient I filled this tooth at once, though I recommended extraction. After cleaning out the root-canals, I filled them with diaphtherin and oil of cloves, and closed the cavity with cement. Two days after I was forced to extract the tooth, when I found the roots curved and the palatine one filled with strong-smelling pus. This tooth, of course, would have needed a very different treatment.

But in spite of this it will be seen that diaphtherin is an antiseptic superior to any one we have had so far, and I should recommend every dentist to try it. It is, however, to be remembered that it attacks steel instruments rapidly on account of the oxyquinolin, wherefore it should be used only with strongly nickel-plated instruments. I have a small spoon, like a spoon-shaped excavator, to introduce it into the cavity. For filling root-canals I take a small piece of platinum wire retained in an ivory handle.

